# Turnip Mosaic Virus Coat Protein Deletion Mutants Allow Defining Dispensable Protein Domains for ‘in Planta’ eVLP Formation

**DOI:** 10.3390/v12060661

**Published:** 2020-06-19

**Authors:** Carmen Yuste-Calvo, Pablo Ibort, Flora Sánchez, Fernando Ponz

**Affiliations:** Centro de Biotecnología y Genómica de Plantas, Universidad Politécnica de Madrid-Instituto Nacional de Investigación y Tecnología Agraria y Alimentaria (CBGP, UPM-INIA), Campus Montegancedo, Autopista M-40, km 38, Pozuelo de Alarcón, 28223 Madrid, Spain; carmen.yus.cal@gmail.com (C.Y.-C.); pablo_ibort@hotmail.com (P.I.); florasanchez@telefonica.net (F.S.)

**Keywords:** VNPs, assembly, eVLPs, mutant, deletion, potyvirus, coat protein

## Abstract

The involvement of different structural domains of the coat protein (CP) of turnip mosaic virus, a potyvirus, in establishing and/or maintaining particle assembly was analyzed through deletion mutants of the protein. In order to identify exclusively those domains involved in protein–protein interactions within the particle, the analysis was performed by agroinfiltration “in planta”, followed by the assessment of CP accumulation in leaves and the assembly of virus-like particles lacking nucleic acids, also known as empty virus-like particles (eVLP). Thus, the interactions involving viral RNA could be excluded. It was found that deletions precluding eVLP assembly did not allow for protein accumulation either, probably indicating that non-assembled CP protein was degraded in the plant leaves. Deletions involving the CP structural core were incompatible with particle assembly. On the N-terminal domain, only the deletion avoiding the subdomain involved in interactions with other CP subunits was incorporated into eVLPs. The C-terminal domain was shown to be more permissive to deletions. Assembled eVLPs were found for mutants, eliminating the whole domain. The C-terminal domain mutants were unusually long, suggesting some role of the domain in the regulation of particle length. The identification of the CP domains responsible for eVLP formation will allow for new approaches to protein stretch replacement with peptides or proteins of nanobiotechnological interest. Finally, specific cases of application are considered.

## 1. Introduction

As a relevant step in the viral life cycle, the assembly of the viral particle is a crucial process. In the case of plant viruses, our present knowledge about the mechanisms involved is rather limited. Considering plant viruses with tubular virions, all of them non-enveloped ss(+)RNA viruses, tobacco mosaic virus (rigid virions) is the most studied and best known [1,2]. Potato virus X (flexuous virions), has also been investigated in this regard [3]. Unsurprisingly, particles of these two viruses are among the most widely used for nanobiotechnological developments based on this knowledge [2,4,5,6,7,8,9], although none of them is able to form complete particles in the absence of a guiding RNA, the viral RNA in the case of natural infections [10,11,12,13].

Conversely, the coat proteins (CP) of other plant viruses are able to give rise to particles in the absence of a guiding RNA, the so-called empty virus-like particles (eVLPs, [14]). In the context of assembly studies, these particles are particularly useful because they represent the outcome of exclusively mutual CP interactions established without the need of RNA. In addition to the fundamental nature of the knowledge about these CP–CP interactions, the information about them in eVLPs also has significant importance in the emerging area of the nanobiotechnological deployment of plant virus-derived nanoparticles [15]. In many instances virion functionalization is not advisable for biosafety reasons [16,17,18], and in other cases the modifications involved in functionalizations abolish virus infectivity [19,20], hence precluding their use. In most cases, these problems can be overcome by using functionalized eVLPs. This applied aspect provides an added value to the CP–CP studies underlying eVLP assembly.

Within plant tubular viruses able to form eVLPs, potyviruses have a prominent position. With members described for most botanical plant families, the potyvirus genus (flexuous virions) contains over one third of all described plant virus species [21,22]. Their detailed structure has been recently solved for three of them by cryo electron microscopy [23,24,25]. The eVLP structure is also available for two of them [24,25]. These structural studies uncovered a left-handed helical array of CP subunits, each of which can be grossly divided in three major domains. The central or core one is a region made of helices highly compacted and the N- and C-terminal domains are long and flexible. The N-terminal one is solvent-oriented, although its most distal part could not be solved for technical reasons. The most distal part of the C-terminal domain has not been resolved either, except for potato virus Y (PVY) [24]. The solved part in the domain is contained within the lumen of the particle. There is some controversy about the global structure of the solved eVLPs. While, in the case of turnip mosaic virus (TuMV), a helical symmetry similar to the virion has been found, in the case of PVY, a non-helical stacked-ring architecture has been proposed.

The detailed structural knowledge about potyvirus virions and eVLPs (collectively referred to as viral nanoparticles (VNPs)) is facilitating their nanobiotechnological exploitation. Thus far, most developments have been carried out in TuMV VNPs. For instance, selected peptides have been genetically fused between the first and second amino acid at the exposed N-terminal region, obtaining VNPs functionalized for different biotechnological applications [19,20,26]. Chemical functionalizations of TuMV VNPs have also been performed by click chemistry [27] or by crosslinking of the VNPs with an enzyme forming nanonets [28]. Structure-based combined approaches to genetic and chemical functionalizations are also possible, thus opening the door to VNPs functionalized with multiple purposes [29]. More work is currently in progress for an increased exploitation of the recently gained TuMV structural knowledge. However, this knowledge is still incomplete mostly because of the uncertainty of the CP structure at its more distal domains. It is not known if these domains are dispensable for particle formation, or the possible interactions they can take place in. This type of knowledge has an important fundamental value and can also serve as the basis for further, still unexplored, nanobiotechnological deployment of the particles.

Until more detailed structural studies of these regions are available, an approach involving deletion mutants of the CP, followed by the examination of their ability to form particles, can provide answers to those questions. Such a study is presented in this work, whose results are also discussed under the perspective of the virus structure.

## 2. Materials and Methods

### 2.1. Construction of Expression Plasmids

For plasmid construction to express different truncated versions of the capsid protein, a previous construct of the expression plasmid pEAQ-HT-Dest1 with the coding sequence of the complete capsid protein, was used [19]. A PCR (GeneAmp^®^PCR System 9700, Applied Biosystems, Foster City, CA, USA) was performed on this plasmid, amplifying the sequence encoding the truncated versions, in addition to the sequences corresponding to the initiation codon, the STOP codon and the sequence in 5’ CACC for directional cloning into the pENTR^TM^/D-TOPO^TM^ vector (ThermoFisher, Waltham, MA, USA). Once in the pENTR vector, the insert was moved to the expression vector pEAQ-HT-Dest1 by Gateway cloning, using the enzyme LR clonase (ThermoFisher), and following the manufacturer’s instructions. Information regarding the cloning process and plasmids appears in Figures S1 and S2.

### 2.2. Production and Purification

For eVLP production, different pEAQ constructs were transformed into *Agrobacterium tumefaciens* LBA4404 for agroinfiltration-mediated transient expression in *Nicotiana benthamiana* plants [30,31]. Plant growth, *Agrobacterium* culture preparation, agroinfiltration, tissue harvesting, and VLP purification were performed as previously described by us [19,26].

### 2.3. Immunoassays

To evaluate the production and accumulation of the viral CP in agroinfiltrated plant tissue, an indirect ELISA was performed using the anti-poty antibody (Agdia, Elkhart, IN, USA), a monoclonal antibody directed against a conserved epitope in potyvirus CPs, which was still present in all the deleted constructs. The plates (Nunc MaxiSorp, ThermoFisher, Waltham, MA, USA) coated with 100 µL plant extract, obtained by homogenizing 1 cm^2^ of agroinfiltrated tissue in 250 µL of extraction buffer (50 mM sodium carbonate buffer, pH 9.6), were incubated overnight at 4 °C. After incubation, the plates were washed intensively and then incubated with the primary antibody, diluted in conjugate buffer (phosphate-buffered saline, pH 7.4, 0.05% Tween 20 (*v*/*v*), 2% Polyvinylpyrrolidone (PVP) 40 (*w*/*v*)) following the manufacturer’s specifications, for 1 h at room temperature. Then, secondary antibodies, diluted 1:1500 in the same buffer, were added and incubated for 1 h at room temperature. The color was developed by an alkaline phosphatase reaction and detected after the addition of *p*-nitrophenylphosphate. Absorbance was measured at 405 nm (TECAN Genios Pro, Männedorf, Switzerland).

### 2.4. Transmission Electron Microscopy (TEM)

The eVLP assembly was assessed by TEM, performed as previously described [20].

## 3. Results and Discussion

**Figure 1:**
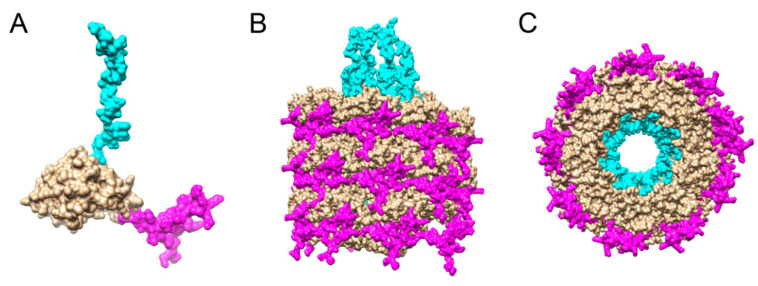
Localization of CP domains in turnip mosaic virus (TuMV). In pink, amino acids 66–97, corresponding to the N-terminal-solved domain (NS), in blue, amino acids 245–272, corresponding to the C-terminal-solved domain (CS). The first sixty-five and the last sixteen amino acids are not represented, corresponding to NNS and CNS regions. (**A**) Monomer. (**B**) Lateral view of the assembled virus. (**C**) Axial view of the assembled virus.

As a first step for deletion design, we located the TuMV CP domains to be interrogated, within the recently published virus structure (PBD ID 6T34 [25]). In this structure, the N-terminal domain spans aa 1–97, the C-terminal covers aa 245–288, and the rest of the protein lies within the core domain (Figure 1). In the Figure, the most distal regions of the domains are not represented (specifically residues 1–65 at the N-terminal domain, and 273–288 at the C-terminal domain), because its structure could not be solved by CryoEM. For short, region 66–97 will be named the N-solved region (NS) and region 245–272 as the C-solved region (CS). Absent regions in the structure will be named as NNS and CNS.

Evaluating the possible implications of the solvent-exposed N-terminal domain in the assembly, the NS region shows an important implication in virion integrity, interacting with the core domain of two other subunits, one located in the same turn (subunit +1) and another one in a different turn (subunit +10). Regarding the NNS region, investigations related to immunogenicity of different CP sequences [32], as well as the assembled particle susceptibility to mild digestion with trypsin [33] indicate that CP first residues are also exposed to the solvent, theoretically without interacting with other subunits. This would give to the DAG motif, located in the most distal region of the NNS domain, a high level of exposure favoring interaction with the aphid stylet for virus spread [34].

At the C-terminal domain, the CS region forms an internal structure around the particle pore, sustained by small and local interactions with upper and lower subunits. According to the available structural model, CS presents an electronegative potential, which could generate repulsion forces between different subunits, thus providing turgor to the inner wall of the particle and contributing to channel formation. Despite the absence of a well-defined CNS structure in the TuMV model, the solved structure of another potyvirus, PVY [24], shows that this domain forms a compact cone-shaped structure at the end of the CP, within the pore lumen. The internal location of this conical-structure, and the poor immunogenicity of the C-terminal domain in comparison with the N-terminal domain [32], are in opposition to the proposed solvent exposure of the CNS region based on trypsin mild digestion [33]. These studies would indicate that the CNS region would present a minimal solvent-exposure and poor interaction with other subunits, presenting a certain level of flexibility in TuMV and other potyviruses [23,25].

**Figure 2:**
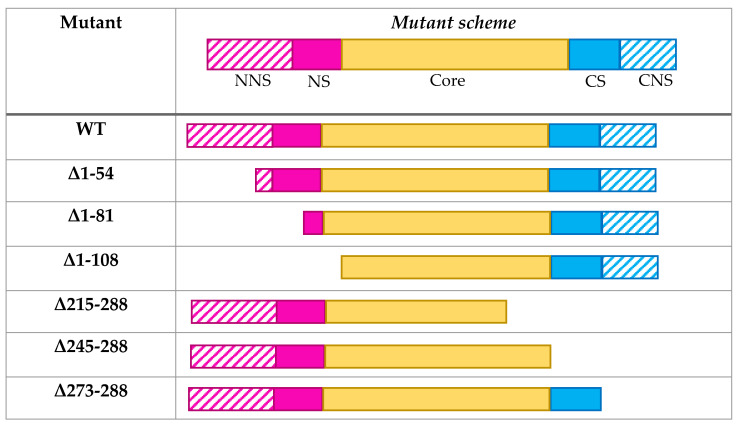
Design of coat protein truncated versions. NNS: CP N-terminal non-solved region (aa 1–65). NS: CP N-terminal-solved region (aa 66–97). CS: CP C-terminal-solved region (aa 245–272). CNS: CP C-terminal non-solved region (aa 273–288). Each of these domains is represented with different colors: NNS (striped pink), NS (solid pink), CS (solid blue), and CNS (striped blue).

To assess the implication of the different domains in particle assembly, CP deletion mutants were designed to generate symmetric constructs: one pair of mutants in NS and CS, another pair in NNS and CNS, and two additional deletions implicating part of the core domain from each end. Previous CP deletion studies in the *Potyviridae* have claimed the implication of regions of different lengths in particle assembly, but these studies were conducted before the structural characterizations, consequently they were not structure-based, with the exception of PVY [24,35,36,37]. In the present work, the structure-based deletions allow to focus on the interactions between structural domains. The deletion mutants and the terminology used are shown in Figure 2. Possible nanobiotechnological applications of the mutant eVLPs are also considered and discussed. Additional information is provided in the Figures S3 and S4.

The first construct is just wild type CP, whose plant-made eVLPs have already shown their usefulness in previous nanobiotechnological applications [19,20]. In this case, TuMV eVLPs-WT (wild-type) show a morphology similar to virion particles, with a helical symmetry and a size range of 700–1000 nm [25]. eVLPs have also been developed in another potyvirus, PVY, through a yeast production system [24]. Unlike TuMV, PVY eVLPS do not retain helical symmetry, but are formed of stacked discs, presenting a greater size dispersion, from 300 nm to 3.5 microns.

**Figure 3:**
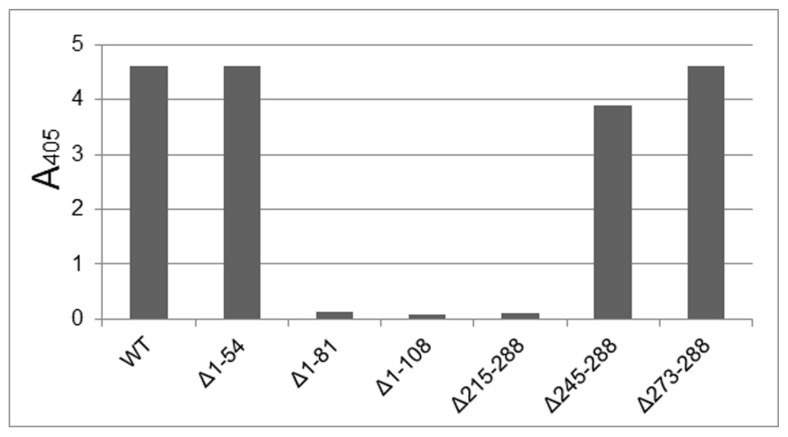
Anti-poty ELISA of protein extracts from leaves agroinfiltrated with the different constructs.

In TuMV deletion mutants, the ability to accumulate protein and assemble particles was evaluated, so as to identify indispensable domains for assembly. Production and accumulation of the CP truncated versions was evaluated by an anti-poty ELISA on agroinfiltrated plant tissue, 12 days after infiltration, for the detection of potyviral CP. The ELISA results are shown in Figure 3.

According to the results obtained, the NNS, CS and CNS regions can be deleted without affecting CP production and accumulation. No accumulation of the protein was found after deleting the NS domain or stretches of the core region. These results indicate that either the truncated protein is not produced adequately, or there is no accumulation due to its rapid degradation. We favor the degradation interpretation and assume that the protein is not capable of forming stable high-ordered structures, i.e., particles with a certain level of assembly, since there is no obvious reason why the deleted protein could not be correctly translated. This interpretation makes sense if we consider the interactions taking place between the core domain and the NS, very important for the assembly of viral particles [23,24,28].

**Figure 4:**
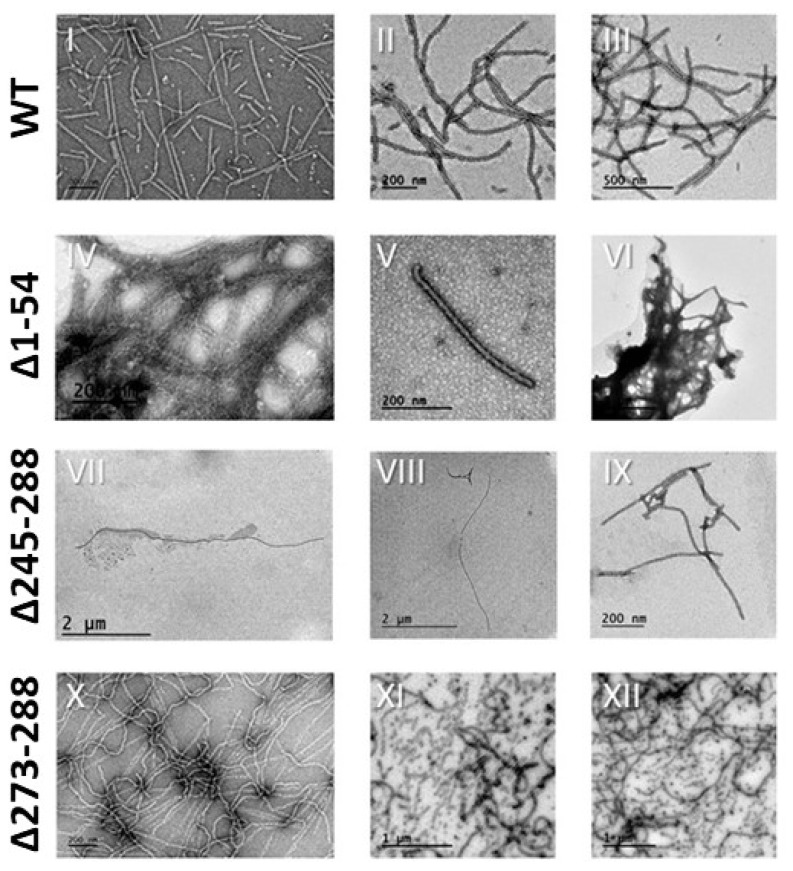
TEM micrographs of purified eVLPs from different constructs. Images I, IV, VII and X were taken right after purification. The rest of the images were taken 4 weeks after purification.

In order to assess expendability of the CS, CNS and NNS regions for particle assembly, constructs WT, Δ1-54, Δ273-288 and Δ245-288, giving a high level of production and accumulation, were purified. All of these constructs gave rise to eVLPs, clearly indicating that the deleted regions do not play an essential role in assembly. Particles were then analyzed by TEM to determine the stability of their structural integrity, as well as possible modifications. Some of the obtained micrographs are shown in Figure 4.

Mutant particles Δ1-54 tend to form visible and insoluble aggregates that remain in suspension briefly before depositing and appear in the micrographs as intricate protein nets (Figure 4, IV and VI). It was possible to locate some isolated nanoparticles (Figure 4, V), but in an extremely low concentration and with a 400–600 nm length, shorter than eVLPs-WT of 700–1000 nm. Previous deletion studies in the *Potyviridae* identified stretches in the N-terminal domain that still allowed assembly, whereas deletions affecting larger parts prevented assembly [24,35,36,37]. Our results with TuMV clearly indicate that it is the specific structural region within the domain that specifies its involvement in assembly. The N-terminal domain length in the *Potyviridae* is highly variable [38,39], thus the mere deletion size is not so informative in this context. The length of the deleted region does not seem to be what determines assembly, but its position relative to the whole structure.

A peculiar characteristic of the Δ1-54 particles is their tendency to form aggregates. Considering the structural position of the NNS region, this portion of the N-terminal domain could, in fact, be considered as a functional ‘spacer’, such that its absence would allow new lateral interactions between individual nanoparticles and induce aggregation into nets. These nanoparticles could be deployed when a large protein accumulation is necessary. This type of application would be similar to that obtained by adding glutaraldehyde to the viral nanoparticles [28], but forming larger aggregates with different characteristics of aggregation and solubility. Some applications include, for example, enzymatic immobilization on a solid phase (the eVLP nets), the slow release of substances from organic materials, or other related applications with highly localized concentrations of substances.

Regarding the C-terminal domain, Δ273-288 eVLPs appear as highly elongated nanoparticles, some of them reaching more than six microns. These long nanoparticles are brittle as shown in Figure 4-VIII, where a very long nanoparticle appears segmented, although most of the nanoparticles were similar to eVLPs-WT, with a length around 700 nm. These results show that the CNS region does not form interactions necessary for assembly. The eVLPs obtained have a diameter similar to eVLP-WT according to measurements made with the electron microscope software, which could indicate that CNS is not relevant in the structural integrity of the viral pore. The structural maintenance of these eVLPs, in which flexuosity and diameter are preserved (although with more length dispersion), in addition to their similar stability to eVLPs-WT, could allow their use as nanoparticles. If future structural studies reveal that the C-terminal domain is exposed to solvents in its most distal region, truncated eVLPs could be functionalized with genetically fused peptides whose activity requires C-terminal exposure. Thus far, genetic fusion applications have been developed only at the N-terminal domain [19,26]. A precise structural characterization would facilitate a much better definition of possible applications of these mutant particles.

Finally, Δ245-288 eVLPs are nanoparticles much longer than WT-eVLPs, with lengths of several microns. Furthermore, these particles are more flexuous, although with a similar diameter to unmodified eVLPs. Over time, these nanoparticles tend to fragment into small segments (Figure 4, XI, and XII). They are, therefore, more brittle than Δ273-288 eVLPs. Because the difference between Δ245-288 deletion with respect to Δ273-288 is the absence of the CS region, the absence of this structural support could imply loss of stiffness. As already mentioned, the CS forms the internal structure of the pore, with a negative charge that could generate repulsion forces, providing turgor to the pore. Without these forces, the subunits would be able to interact among themselves, and somehow generate very long particles, but with a greater tendency to fragment into small sections, less than 100 nm long. Nanoparticles obtained with a similar deletion in PVY do not show these differences in terms of length, flexibility and resistance. In PVY the resulting particles are very similar to WT, with the same symmetry of stacked disks and similar characteristics of length, flexibility and hardness [24]. Considering the specific structural characteristics of these TuMV nanoparticles, they could be applied when no long-term stability is required, such as release of different molecules attached to the subunits.

Both TuMV C-terminal mutants (Δ245-288 and Δ273-288) are unusually long compared to WT-eVLPs. This observation suggests that the domain (especially the CNS, absent in both mutants) could have a role in the regulation of the length of the assembled particles. In the case of PVY eVLPs (both WT and C-terminal deleted), the particles show significant differences compared to those of TuMV and, as described above, the deletions at the C-terminal region do not increase their length. For PVY, the whole C-terminal domain has been solved, showing that it is fully luminal and, in the case of virions, involved in interactions with the viral RNA. This type of precise structural information is not available for TuMV. The differential results obtained for eVLPs of viruses so similar could be the reflection of subtle differences between different potyviruses in this region. Again, more structural studies should shed light about this point.

All the results obtained show that the NNS and CNS regions are not essential for CP self-assembly, since they do not seem to generate indispensable interactions for particle formation. The implication of these domains in the infectious process of potyviruses and viruses with similar shape was previously studied [24,33,35,37,40,41]. The results of these studies are more related to infectivity and movement than to strict assembly, showing that the N-terminal domain is essential for TuMV transmission, while everything indicates that the C-terminal is mostly involved in movement. A more detailed structural study and the development of different mutants will allow for specific VNP designs for different biotechnological applications. TuMV VNPs appear again as versatile alternatives for the development of biotechnological tools for numerous fields, from industrial processes to biomedical theranostics, if based on an in-depth knowledge of their basic characteristics regarding particle assembly for specific VNP development.

## Supplementary Materials

The following are available online at https://www.mdpi.com/1999-4915/12/6/661/s1, Figure S1: PCR Products scheme; Figure S2: Plasmids; Figure S3: CP domains distribution; Figure S4: Schematic representation of deletion mutants.
