# Supplementary material for: Turnip Mosaic Virus Coat Protein Deletion Mutants Allow Defining Dispensable Protein Domains for ‘in Planta’ eVLP Formation
Source: Viruses. 2020 Jun 19;12(6):661. doi: 10.3390/v12060661 (PMC7354486; doi:10.3390/v12060661)
Supplement: Supplementary file 1 [file viruses-12-00661-s001.pdf]

viruses

MDPI

Supplementary material

Turnip mosaic virus coat protein deletion mutants allow defining dispensable protein domains for 'in planta' eVLP formation

Carmen Yuste-Calvo [1], Pablo Ibort [1,§], Flora Sánchez [1] and Fernando Ponz [1,*]

[1] Centro de Biotecnología y Genómica de Plantas, Universidad Politécnica de Madrid - Instituto Nacional de Investigación y Tecnología Agraria y Alimentaria (CBGP, UPM-INIA), Campus Montegancedo, Autopista M-40, km 38. Pozuelo de Alarcón, 28223 Madrid, Spain.

[§] Present address: R&D Microbiology Department. Koppert Biological Systems. Berkel en Rodenrijs. The Netherlands

* Correspondence: fponz@inia.es

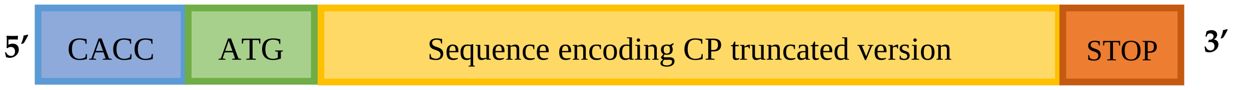

Figure S1. PCR Products scheme. All PCR products contain the sequence CACC for directional cloning, the ATG to open the ORF, the sequence encoding the different CP truncated versions and a sequence encoding the amber stop codon to close the ORF.

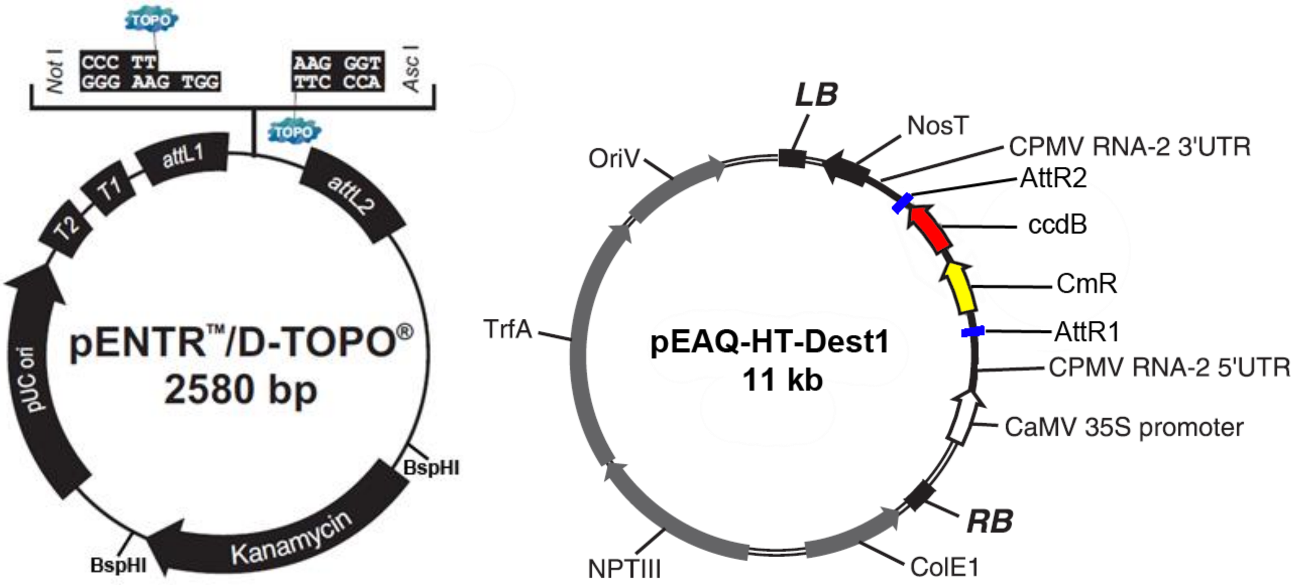

Figure S2. Plasmids. Left: PENTR™/D-TOPO®. It shows the GTGG sequence for directional cloning of the PCR product, flanked by the restriction sites *Not I* and *Asc I*, terminators T1 and T2, the origin of replication pUC, the sequences for cloning Gateway attL1 and attL2 and the target-flanked kanamycin resistance gene for the restriction enzyme BspHI. Right: pEAQ-HT-Dest1. The region involved in Gateway recombination is between the UTR regions. The chloramphenicol resistance gene (CmR) is shown in yellow, the cell proliferation control gene (cddB) in red, both flanked by attR regions (blue), which recombine with the AttL regions in the pENTR vector.

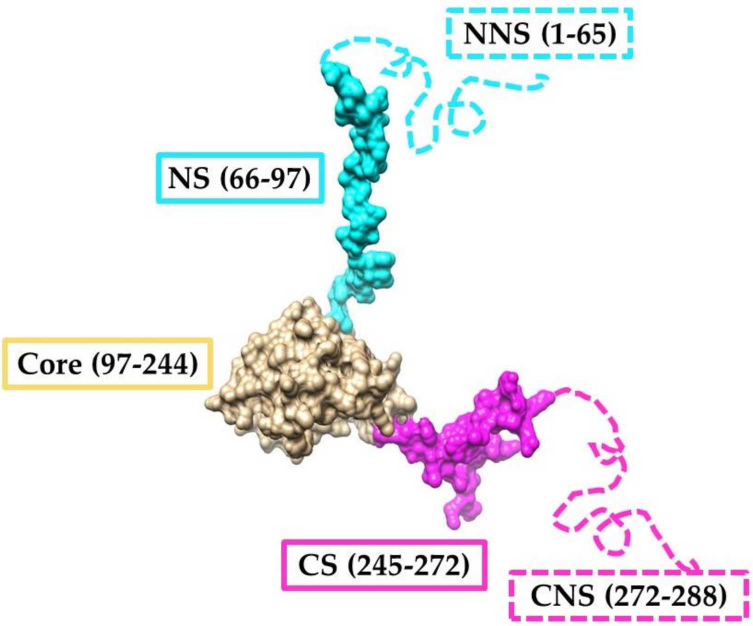

Figure S3. CP domains distribution. Schematic representation of the reported CP regions, both those solved by CryoEM, and the non-solved. These are represesented as discontinuous lines. Amino acids involved on each region are also described.

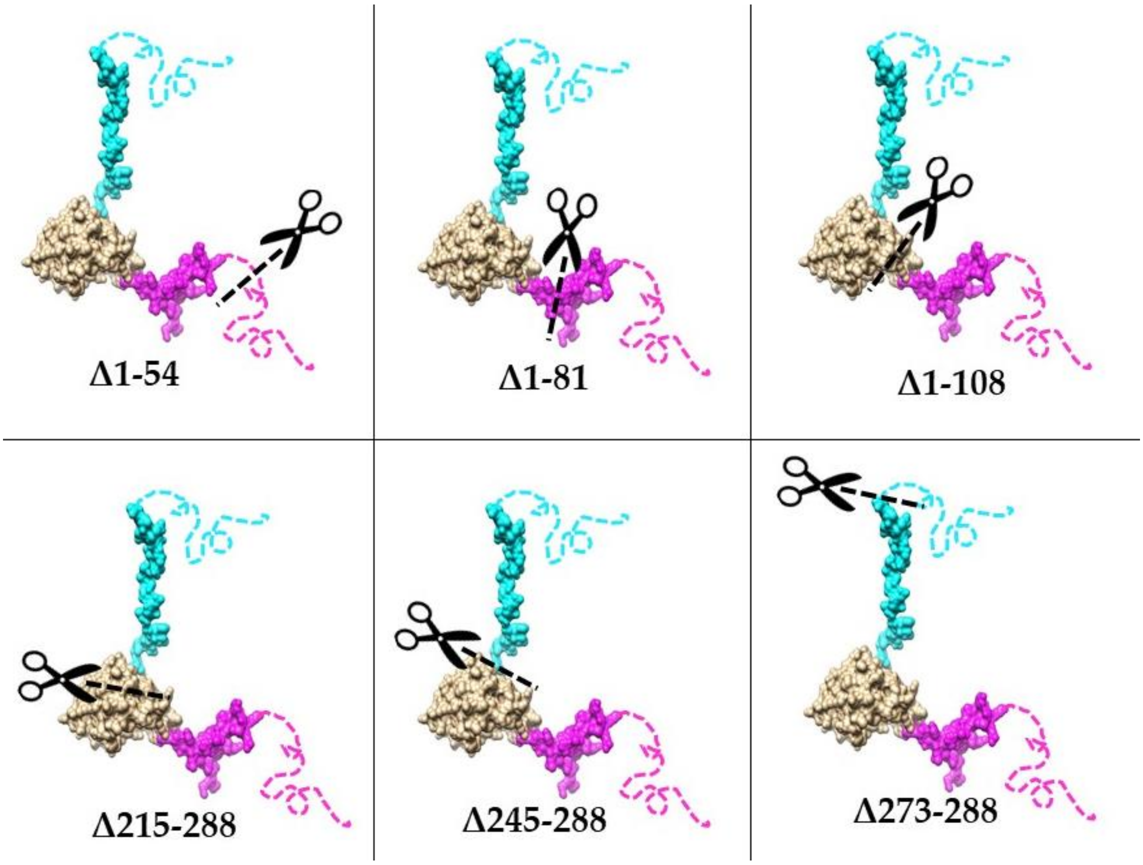

Figure S4. Schematic representation of deletion mutants.

© 2020 by the authors. Submitted for possible open access publication under the terms and conditions of the Creative Commons Attribution (CC BY) license (http://creativecommons.org/licenses/by/4.0/).
